# Ambulatory Tasks and Journeys: A Framework for Free-Living Behaviour

**DOI:** 10.3390/s26061754

**Published:** 2026-03-10

**Authors:** Craig Speirs, Le Wei, Matthew Ahmadi, Mark Hamer, Emmanuel Stamatakis, Malcolm Granat

**Affiliations:** 1 PAL Technologies Ltd., Glasgow G4 0TQ, UK; craig.speirs@strath.ac.uk; 2 Department of Computer and Information Sciences, University of Strathclyde, Glasgow G1 1XH, UK; 3 Mackenzie Wearables Research Hub, Charles Perkins Centre, The University of Sydney, Sydney, NSW 2050, Australia; lwei6596@uni.sydney.edu.au (L.W.); matthew.ahmadi@sydney.edu.au (M.A.); emmanuel.stamatakis@sydney.edu.au (E.S.); 4 School of Health Sciences, Faculty of Medicine and Health, The University of Sydney, Sydney, NSW 2050, Australia; 5 Division of Surgery and Interventional Science, Faculty of Medical Sciences, University College London, London WC1E 6BT, UK; m.hamer@ucl.ac.uk; 6 School of Health and Society, University of Salford, Salford M6 6PU, UK

**Keywords:** free-living physical behaviour, accelerometry, physical activity, event-based classification, BCS70, activPAL

## Abstract

Background: Standard accelerometer summaries obscure meaningful differences in how people move while upright. We introduce an operational two-class Ambulatory Behaviour Framework that separates Ambulatory Tasks—periods of standing and short continuous stepping bouts (<1 min) that are indicative of activity in a single locus—from Ambulatory Journeys—long continuous stepping bouts (≥1 min) that are indicative of movement between locations. Methods: We analysed thigh-worn activPAL3 data from 3545 participants in the age-46 sweep of the 1970 British Cohort Study (24,815 valid monitor-days). Event-based algorithms grouped upright events and classified them as an Ambulatory Task or Journey; linear models examined associations with sitting time and differences by sex and BMI. Results: Mean upright time averaged 6.50 h day^−1^; Ambulatory Tasks dominated (5.91 h; 90.6% of upright exposure), whereas Ambulatory Journeys contributed 0.61 h (9.4%). Each additional hour of Ambulatory Tasks corresponded to 0.61 h less sitting (β = −0.61 h; 95% CI: −0.63 to −0.61), while an extra hour of Ambulatory Journeys displaced only 0.04 h of sitting (β = −0.04 h; 95% CI: −0.044 to −0.039). Women accumulated significantly more time in Ambulatory Tasks and less sitting time than men. Both upright behaviours declined with increasing BMI. Conclusions: Ambulatory Tasks substantially replace sitting time, whereas Ambulatory Journeys leave sitting essentially unchanged. Interventions to displace sitting should concentrate on fostering frequent, brief, context-embedded tasks throughout the day. This novel framework yields interpretable, sensor-agnostic metrics to target behaviour change and standardise reporting of free-living mobility.

## 1. Introduction

Current frameworks for classifying physical behaviour predominantly categorise activity based on broad metrics, such as total step counts [1] or bout durations, often identifying clear thresholds for sedentary, light, moderate, and vigorous intensities [2]. Although convenient, this oversimplified classification approach neglects important qualitative differences in stepping behaviours.

Increasingly, studies classify stepping not only by total volume but also by the intensity and pattern of movement [3]. One common approach is to define cadence bands—ranges of stepping rate in steps per minute—as proxies for intensity [4]. In parallel, frameworks often distinguish stepping bouts by duration: short bursts of steps are treated differently from sustained periods of stepping. A threshold such as 1 min of continuous stepping can be used to define a “walking bout,” so that brief stepping bouts might be considered incidental, while longer bouts signify meaningful ambulatory activity [5]. This approach has proven especially useful to determine walking within the home, where stepping typically consists of brief bouts. For instance, Speirs et al. [5] reported that stepping occurring within a home environment tends to involve shorter stepping bouts (often under one minute) compared to outdoor stepping during community or recreational activities, while shorter periods of straight-line stepping are typically associated with home-based activities [6]. Even among older adults, nearly one-third may not achieve any walking bout longer than 6 min during a week in normal daily life [7], a reflection of how uncommon prolonged continuous walks can be in unrestricted settings. While cadence bands and bout duration cut-offs provide a practical way to classify stepping behaviour, these schemes do not account for standing periods that might be part of the stepping activity and are inherently part of a person’s ambulatory behaviour. This limitation underscores the need for a more inclusive framework in ambulatory behaviour research, as a more nuanced approach to classifying stepping behaviour would allow for the development of interventions that target the aspects of physical behaviour that are most amenable to change.

In addition to stepping, recent evidence shows that not all standing is the same [8]. A person standing perfectly still (e.g., waiting in a queue or standing at a desk looking at a screen without moving) versus someone fidgeting or shifting weight while standing can have different physiological and behavioural implications. There have been suggestions to better characterise standing intensity in activity monitoring [9], separating passive standing (essentially motionless upright posture, typically ~2 METs or less) from active standing (standing with subtle movements that slightly elevate energy expenditure). To detect this distinction, Kowalsky et al. used acceleration-variance features: a standing period with virtually no acceleration fluctuation indicates passive standing, whereas higher signal variance marks active standing. Classification approaches, including machine learning models, have been applied to thigh-worn sensor data to differentiate these states [10]. These methods aim to capture postural dynamics that go beyond the coarse sit/stand dichotomy. However, these approaches have important limitations. They rely on heuristic cut-offs or trained models to classify active and passive standing, which means that determining how much movement qualifies as “active” often depends on arbitrary or dataset-specific criteria. In addition, current activity classification frameworks (and devices like standard accelerometer algorithms) do not integrate this distinction—they typically label any upright, non-walking time simply as “standing”.

Although passive and active standing can now be distinguished in research datasets, this capability remains siloed from any overarching behaviour model. The resulting gap prevents us from fully characterising upright behaviour; in particular, short-duration stepping bouts embedded within standing tasks—common in household activities—often go unrecognised. Consequently, current classification systems systematically underestimate both ambulatory energy expenditure and the functional mobility embedded in everyday tasks. A comprehensive Ambulatory Behaviour Framework is therefore required, one that integrates these short stepping bouts into the continuum of ambulatory activity.

To address these limitations, we propose a novel Ambulatory Behaviour Framework—a taxonomy that captures nuanced combinations of standing and stepping. We distinguish two categories:

Ambulatory Tasks (ATs)—standing behaviour punctuated by short (<1 min) bouts of stepping, which would typically be confined to a single locale (e.g., moving between a desk and a printer or within a kitchen).

Ambulatory Journeys (AJs)—prolonged periods (≥1 min) of walking, running, or cycling that indicate that the person is moving between locales (e.g., corridor walking, campus transfers, outdoor travel), potentially retuning to the starting location.

This study had four aims:

1. Operationalise the Ambulatory Behaviour Framework in free-living accelerometry.
2. Quantify daily time spent on Ambulatory Tasks and Ambulatory Journeys and describe sex differences.
3. Examine how each behaviour relates to each other and to sitting time.
4. Examine the relationship between time spent on different categories of ambulatory behaviours and participant characteristics.

## 2. Materials and Methods

### 2.1. Study Design and Participants

This analysis draws on accelerometer data collected during the age-46 sweep (2016–2018) of the 1970 British Cohort Study (BCS70), a long-running population study that originally recruited ≈17,000 UK births from a single week in 1970 [11]. Of the 6492 participants who consented to thigh-worn monitoring, 3545 returned ≥7 days of valid data and constitute the analytic sample. The sweep received NHS Research Ethics Committee approval (South-East Coast, Brighton & Sussex); written informed consent was obtained from all participants.

### 2.2. Device, Wear Protocol and Primary Processing

Participants were fitted with a waterproofed triaxial activPAL3-micro (PAL Technologies, Glasgow, UK) on the mid-anterior thigh by trained nurses and asked to wear the device continuously for seven days before returning it by post [12]. Raw datx files were batch-processed in PALbatch v9.1 using the GHLA algorithm, yielding an event file in which every uninterrupted bout of sitting/lying, standing, stepping or cycling is a single record. Device non-wear was flagged automatically; a day was deemed valid if it had 24 h of valid physical activity data. In line with our previous research investigating this cohort [5], participants were included in the analysis if they had at least seven valid days of valid physical activity data.

### 2.3. Event-Based Behaviour Classification

Event files were imported to R (v 4.3.3) and, an open implementation that refines activPAL posture and activity labels and merges adjacent stride events into single stepping events [13]. Consecutive standing and stepping events bounded by any siting or lying posture were grouped into upright containers following the event-based paradigm [14].

The present study extends that paradigm by classifying periods of upright activity into one of two mutually exclusive ambulatory movement classes:

- Ambulatory Tasks (ATs)—all periods of standing and stepping bouts that are shorter than one minute (capturing in-locus movements such as walking from room to room in a house).
- Ambulatory Journeys (AJs)—periods of continuous stepping or cycling longer than one minute, denoting purposeful, between-locus travel.

These operational rules are consistent with the indoor-community-recreation stepping heuristic validated in earlier activPAL work and with broader event-based analyses of free-living behaviour. Sitting behaviour (SB) was defined as any sitting event, including seated transport in a motor-vehicle.

### 2.4. Derivation of Daily Metrics

For each valid day the following variables were calculated:

- Total time spent on ATs, AJs and SB.
- Number of steps/day; steps accumulated during ATs and AJs.
- Proportion of upright time attributable to ATs and AJs.
- AT: AJ ratio as an indicator of the day’s movement context.

Daily summaries were averaged across the seven-day monitoring period to give participant-level means, preserving within-participant variance by also retaining day-level records for multilevel modelling.

### 2.5. Statistical Analysis

All analyses were conducted in R (v 4.3.3) [13]. Descriptive statistics (medians ± IQR, etc.) summarised the distributions of AT and AJ bout lengths, cadences and temporal profiles. Relationships between time spent on our ambulatory behaviours (ATs and AJs) and time sitting were examined using linear regression models. We tested for sex-specific differences in behaviour time using non-paired t-tests. We carried out ANOVA to identify if there was a significant difference in time spent on different classes of ambulatory behaviours across BMI classifications. Where significant differences were identified, Tukey’s HSD test was used to identify the BMI classifications that had significant differences in activity time. The Freedman–Diaconis rule was used to calculate bin width based on daily time spent on our classes of behaviour [15]. The values were rounded to nearest 5 min to produce sensible charts. A sensitivity analysis was conducted using differing stepping duration thresholds for AJs to test the robustness of our observations.

## 3. Results

### 3.1. Characteristics

Of the 6492 BCS70 participants fitted with an activPAL, 3545 provided the protocol-required ≥7 valid days, yielding 24,815 monitor-days for analysis. Mean wear-time was 24 h ± 0 h, with a mean daily step count of 9210 ± 4676 steps. We observed substantial variation in time spent on Ambulatory Journeys. A summary of the ambulatory behaviour characteristics of the population is given in Table 1.

### 3.2. Daily Exposure in Ambulatory Tasks and Ambulatory Journeys

**Figure 1:**
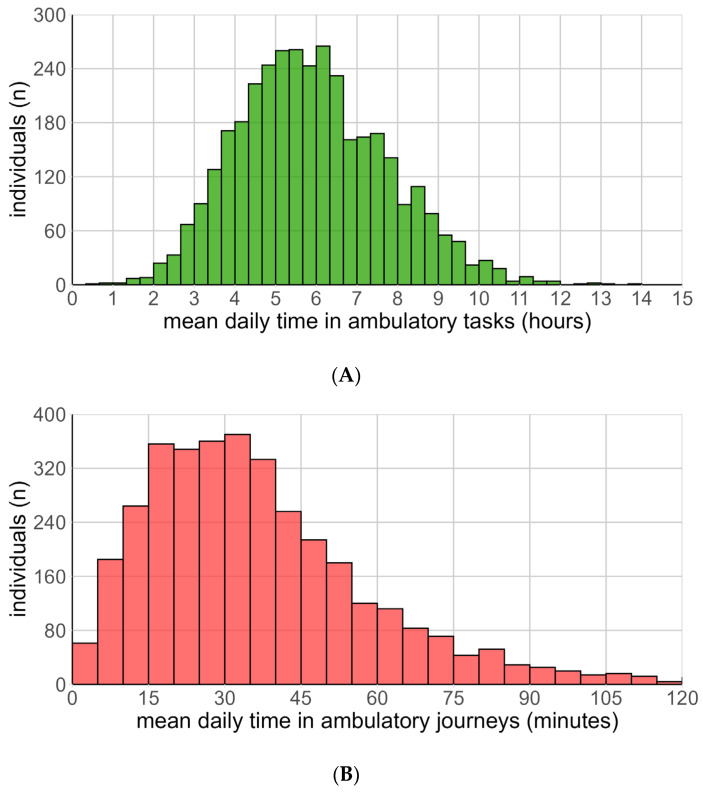
(**A**): Distribution of per-individual mean daily time spent on Ambulatory Tasks. (**B**): Distribution of per-individual mean daily time spent on Ambulatory Journeys.

Participants spent the majority of their upright time on Ambulatory Tasks (ATs). Mean AT time was 5.91 h ± 2.41 h (median = 5.62 h, IQR = 4.11–7.49), representing 24.6% of wear-time (Figure 1A). The distribution was approximately normal (skew = 0.51, kurtosis = 0.01). We found that <9.7% of days recorded less than 3 h of ATs, with 6.1% of days recording more than 10 h of ATs.

Daily time spent on Ambulatory Journeys (AJs) was shorter and had a right-skewed distribution (skew: 1.96, kurtosis: 8.66) (Figure 1B). Participants averaged 36.9 ± 33.6 min spent on AJs (median = 28.0 min), equal to 2.6% of wear-time. Over half of the cohort (58.5%) accumulated less than 35 min of AJs, while 3.1% recorded no AJs on an average day.

**Figure 2:**
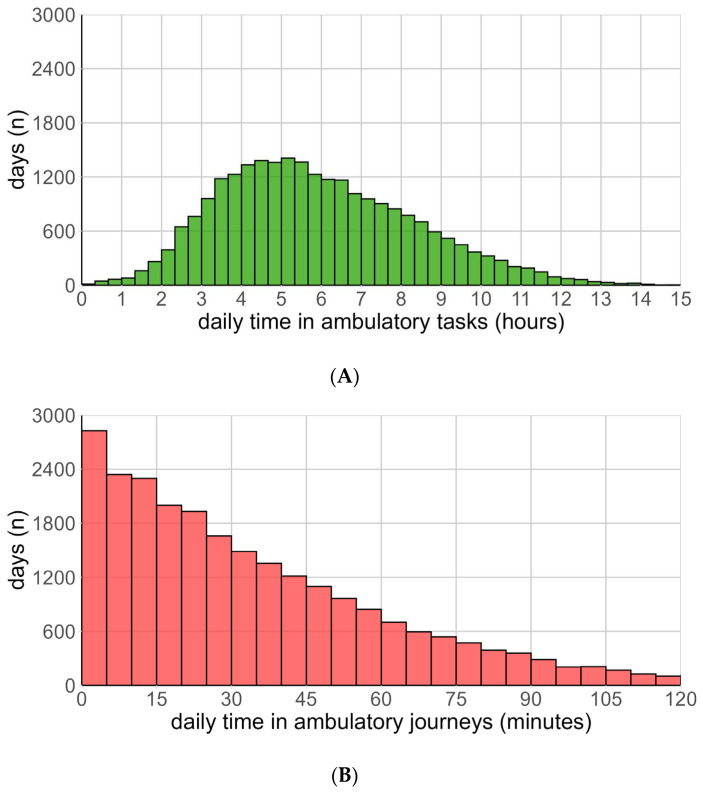
(**A**): Distribution of daily time spent on Ambulatory Tasks. (**B**): Distribution of daily time spent on Ambulatory Journeys.

At a day-level resolution (Figure 2A) ATs remained tightly clustered around 5–6 h; AJs displayed frequent zero-journey days and a long tail of high-journey days, confirming its episodic nature (Figure 2B).

When the daily threshold for classifying stepping bouts as AJs was reduced from 60 s to 45 s, AT time decreased by 7.9 min to 5.78 h ± 2.36 h. Setting the threshold to 75 s increased AT time by 4.8 min to 5.99 h ± 2.43 h (Supplementary Tables S1 and S2).

### 3.3. Relationships Between ATs, AJs and Sitting Time

Mean daily sitting time was 9.16 h ± 2.82 h (median = 9.14, IQR = 7.20 to 11.05).

**Figure 3:**
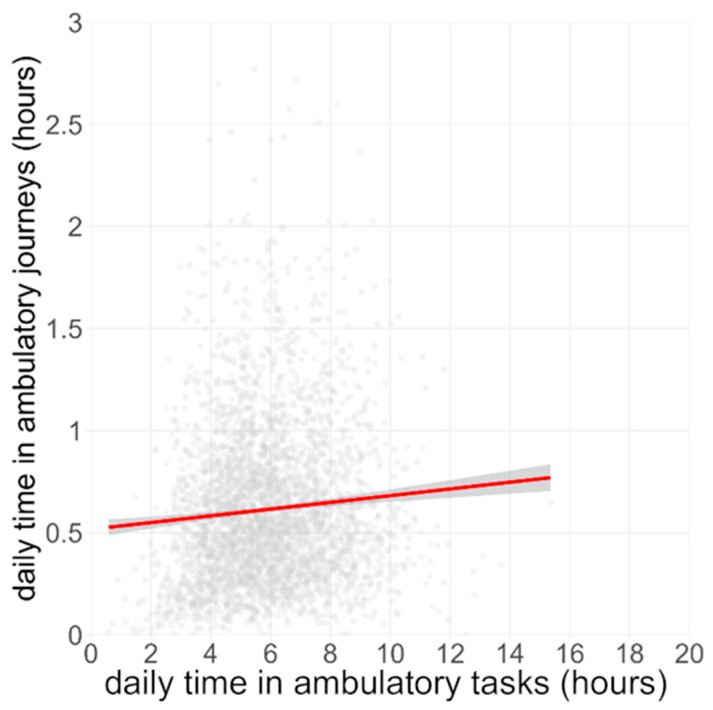
Relationship between Ambulatory Tasks and Ambulatory Journeys. Each grey dot represents a single valid day from the BCS70 cohort (n ≈ 24,800 day-records). The red line shows the ordinary-least-squares fit; the shaded band is the 95% confidence interval.

We observed a weak positive association between daily AJ time and AT time (Figure 3; β = +0.017 h AJs per 1 h ATs, 95% CI 0.014–0.019, R^2^ = 0.005, *p* < 0.01), indicating that days that were richer in brief in-locus movement tended, on average, to include slightly more journey time.

**Figure 4:**
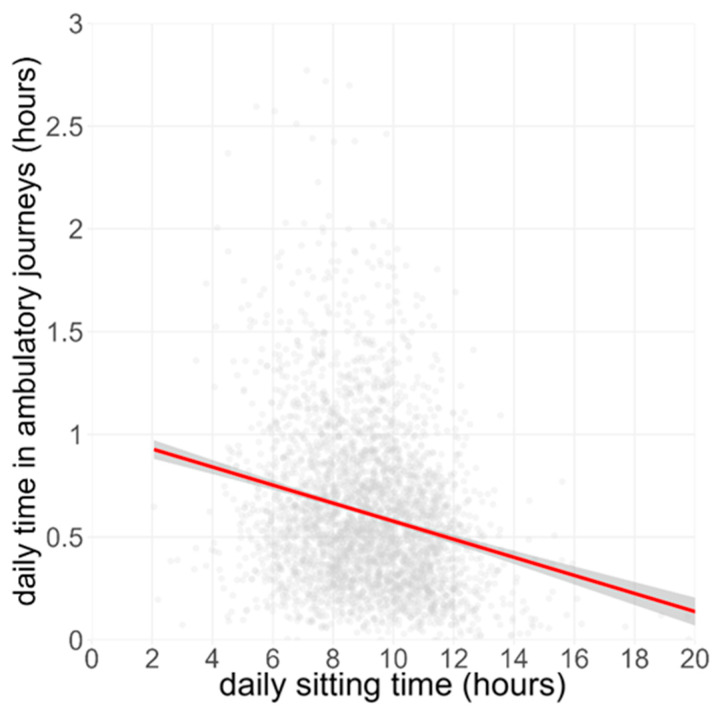
Relationship between sitting time and Ambulatory Journeys. Each grey dot represents a single valid day from the BCS70 cohort (n ≈ 24,800 day-records). The red line is the ordinary-least-squares regression with 95% confidence band (grey).

AJs were modestly inversely related to SB (Figure 4; β =−0.04 h AJ per 1 h SB, 95% CI −0.044 to −0.039, R^2^ = 0.04, *p* < 0.001), but the slope was much shallower than that for ATs, indicating a modest but significant inverse relationship between long bouts of sitting and purposeful, between-locale locomotion.

**Figure 5:**
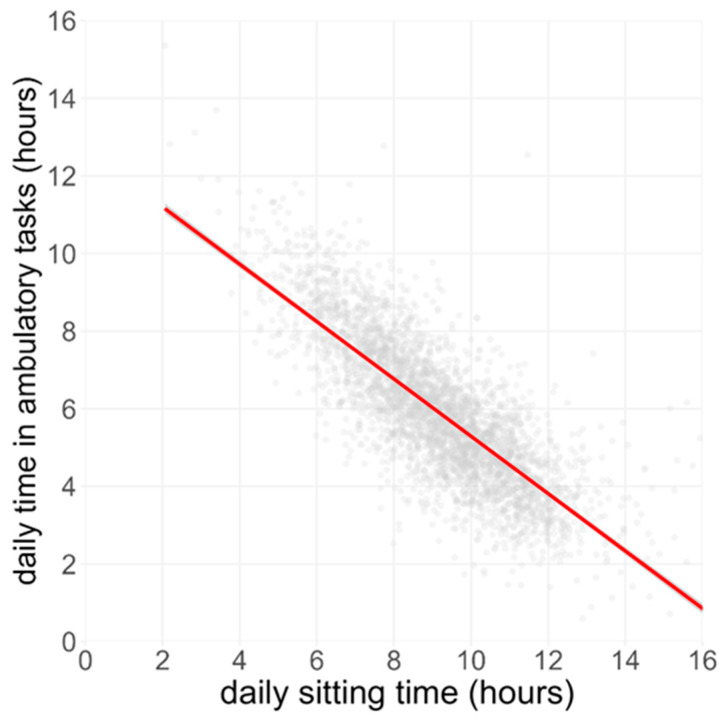
Relationship between sitting time and Ambulatory Tasks. Each grey dot represents a single valid day from the BCS70 cohort (n ≈ 24,800 day-records). The red line is the fitted ordinary-least-squares regression (β ≈ −0.62 h AT per 1 h sitting; R^2^ = 0.52, *p* < 0.001).

Ordinary-least-squares regression across 24,829 days showed a strong inverse association between time spent on ATs and SB (β = −0.61 h AT per 1 h SB, 95% CI −0.63 to −0.61, R^2^ = 0.52, *p* < 0.001; Figure 5), illustrating that individuals who spend more time sitting accumulate markedly less time spent on Ambulatory Tasks.

### 3.4. Relative Contribution of ATs and AJs to Upright Exposure and Sitting Displacement

Across all days, total upright time averaged 6.50 h day^−1^. ATs accounted for a median duration of 5.91 h (≈90.6% of upright time), while AJs accounted for 0.61 h of activity (≈9.4%), giving an average AT:AJ ratio of 9.7. The median per individual AT:AJ ratio was 10.7 (IQR = 6.8 to 17.5).

### 3.5. Sex and BMI Differences in ATs, AJs and Sitting

Across all days, on average males spent 43 min more time sitting (t(24,070) = 20.0, *p* < 0.05) and 3 min on Ambulatory Journeys (t(23,562) = 7.6, *p* < 0.05) compared to females, who on average spent 23 min more time undertaking Ambulatory Tasks (t(24,248) = −12.6, *p* < 0.05).

For BMI, individuals with a BMI classified as healthy (18.5–24.9 kg/m) spent less time sitting and more time undertaking Ambulatory Tasks and Ambulatory Journeys compared to other BMI classifications.

For Ambulatory Tasks, the overweight (25–29.9 kg/m^2^) groups spent significantly more time on ATs than the obese (30–39.9 kg/m^2^) groups. The overweight group also spent significantly more time on ATs than the underweight, obese and morbidly obese (>40 kg/m^2^) groups, while the obese group spent significantly more time on ATs than the morbidly obese group. No other differences in sitting time were observed between the other BMI classifications.

### 3.6. Summary

Quantitatively, ATs comprised approximately 5.9 h of the 24 h movement profile and displaced ~0.61 h of sitting time per additional hour accumulated, whereas AJs contributed only 37 min and displaced SB at a much lower rate (≈2.4 min SB per 1 h AJ). These data suggest that increases in daily mobility and reductions in sedentariness in mid-life adults are driven predominantly by frequent, in-locus movements rather than by longer, more purposeful journeys.

## 4. Discussion

This study set out to operationalise the new Ambulatory Behaviour Framework in a large, nationally representative mid-life cohort and to establish how its two movement classes—Ambulatory Tasks (ATs) and Ambulatory Journeys (AJs)—relate to each other and to sitting behaviour (SB). Three clear findings emerged. First, ATs were the dominant upright behaviour, accounting for over 90% of all upright time, while AJs contributed only 9%. However, there were a significant number of days, accounting for over 10% of days, with less than five minutes of AJs while undertaking large quantities of ATs (4.8 h). Second, almost the entire displacement of sitting time was explained by additional ATs; each extra hour of ATs was associated with almost an hour less of SB, whereas the same increment in AJs reduced SB by only a few minutes. Third, although ATs and AJs were positively correlated, the relationship was weak, confirming that frequent in-locus tasks and occasional between-loci journeys meet different mobility needs.

Our findings raise a question as to why Ambulatory Tasks tend to be the predominant behaviour that displaces sitting? We conjecture that AT bouts are typically embedded in household or occupational routines like preparing food, tidying, and fetching documents and are therefore triggered by contextual cues that arise immediately after or even during periods of sitting. Replacing a seated spell with a small episode of upright movement requires no planning and minimal time commitment; consequently, ATs provide an efficient behavioural “opportunity cost” to reducing prolonged sitting. Physiologically, even low-cadence micro-stepping bouts elicit repeated muscle-pump activation in the lower limbs, increases skeletal-muscle glucose uptake, and elevates energy expenditure by ~0.3–0.5 METs above passive standing. In contrast, AJs may indicate purposeful, between-locale activities that must be scheduled and often occur outdoors; they draw on different motivational constructs (intention, time availability, weather) and tend to be largely uncoupled from the frequency of seated bouts. This additional constraint related to this category of activity may account for the presence of many days where little to no Ambulatory Journeys are undertaken. This behavioural asymmetry may explain why greater AT time is associated with less sitting behaviour, whereas AJs exhibit only a weak inverse relation.

While we observed a decrease in AT and AJ time as BMI diverged from the reference healthy range, our analysis is unable to state the directionality of this relationship. Given that weight has a complex interaction with a range of factors, including dietary intake, physical activity and underlying health, we suggest that AT and AJ times provide a context about a range of factors, including occupation class and general health, that are associated with an individual’s weight and consequently their BMI.

As our proposed framework is based on a stepping duration threshold to classify Ambulatory Tasks and Ambulatory Journeys, we carried out a sensitivity analysis to test the robustness of the framework. Across the population, reducing the threshold for classifying bouts of stepping as Ambulatory Journeys from 60 s to 45 s resulted in an average of 7.9 min of Ambulatory Tasks being reclassified as Ambulatory Journeys, while increasing the threshold to 75 s resulted in an additional 4.8 min of stepping being classified as Ambulatory Tasks. In both instances, the differences in Ambulatory Tasks and Ambulatory Journey time between participants’ sex and BMI group were retained when using different thresholds for Ambulatory Journeys (Supplementary data Table S1).

Our results extend earlier BCS70 analyses that linked total step count to posture composition without distinguishing stepping contexts. Speirs et al. reported that upright time averaged 6 h per day in a population of working age adults and that short periods of stepping predominated at lower activity levels, but the qualitative heterogeneity of those steps could not be assessed under the previous taxonomy. The present event-based classification reveals that most of those “indoor steps” form short, functionally meaningful tasks (<1 min) rather than truncated segments of longer walks.

Likewise, prior work showing that maximum free-living stepping performance resides in a small number of intense bouts is complemented here by evidence that sustained bouts (>1 min) are uncommon in everyday life and may add little to daily energy expenditure. Our finding that ATs replace SB far more effectively than AJs aligns with research indicating that breaking prolonged sitting with brief upright breaks confers cardiometabolic benefit and supports calls to treat standing and very slow ambulation as a continuum of light-intensity activity rather than classifying standing as a sedentary posture.

The thigh placement of the activPAL gives it high sensitivity for detecting posture transitions and short stepping bouts, making it well suited to the present taxonomy. Our AT threshold (stepping bout of less than one minute) was chosen a priori to capture the modal duration of in-locus movement observed in earlier event log analyses; sensitivity checks confirmed that using 30 or 120 s thresholds did not materially alter the AT–SB slope. Nevertheless, AJs were defined solely by bout length; future work could refine this by integrating GPS, turning data derived from a magnetometer or gyroscope, context labels, or cadence cut-points to confirm between-loci intent. This approach would also allow us to identify stride-specific characteristics associated with Ambulatory Journeys that could be used to improve the accuracy of our existing framework.

Strengths include the large, population-based sample, continuous 24 h monitoring, and use of a validated event-based algorithm. As the Ambulatory Behaviour Framework is based on identifying periods of sitting, standing and stepping, this approach can be operationalised using wearable sensors at wear locations where the robust identification of these behaviours is possible. Limitations are the observational design, possible residual confounding (e.g., occupational sitting demand), and reliance on a single week of recording at mid-life. We also lacked contextual information to identify micro-loci within a room, so some very short journeys may have been misclassified as tasks.

## 5. Conclusions

This study outlined a novel framework that uses standing and stepping behaviour to distinguish between two classes of upright behaviour, Ambulatory Tasks (ATs) and Ambulatory Journeys (AJs). Time spent on ATs was around 6 h per day (approximately total 90% of upright time), compared with 0.6 h per day for longer stepping found in AJs. Our study found that decreases in sitting time are primarily driven by increased time spent on Ambulatory Tasks, which comprise standing and short periods of stepping. We also observed that women accrued more AT time and less sitting than men, and both upright behaviours tended to be lower with higher BMI, quantifying the real-world context in which most upright time occurs.

This finding could be used within public health interventions to reduce volumes of sitting by emphasising classes of behaviour that have been shown to displace periods of sitting. This framework provides an approach to classifying upright activity based on behavioural factors that can provide insights that are difficult to obtain using traditional step-count-based frameworks and can also provide policy-relevant outcomes for trials, surveillance and clinical translation.

## Figures and Tables

**Table 1:** Baseline Population Characteristics (mean and SD reported). *—value is significantly different from the reference group.

	Individuals (n)	Ambulatory Tasks (Hours)	Ambulatory Journeys (Minutes)	Sitting (Hours)
**All**	3545	5.91 (2.41)	36.9 (33.6)	9.16 (2.82)
**Sex**				
Male (Reference)	1669	5.70 (2.44)	38.3 (35.5)	9.54 (2.88)
Female	1876	6.09 (2.36) *	35.3 (31.7) *	8.83 (2.71) *
**BMI Group**				
Healthy (Reference) (18.5–24.9 kg/m^2^)	1140	6.16 (2.36)	40.2 (35.5)	8.86 (2.70)
Overweight (25–29.9 kg/m^2^)	1352	5.84 (2.39) *	37.8 (33.8) *	9.25 (2.81) *
Obese (30–39.9 kg/m^2^)	886	5.73 (2.40) *	32.8 (30.7) *	9.36 (2.86) *

## Data Availability

The data that support the findings of this study are available from https://www.ukdataservice.ac.uk/. Restrictions apply to the availability of these data, which were used under licence for this study.

## Supplementary Materials

The following supporting information can be downloaded at https://www.mdpi.com/article/10.3390/s26061754/s1: Table S1: Baseline Population Characteristics with threshold for Ambulatory Tasks set at 45 s; Table S2: Baseline Population Characteristics with threshold for Ambulatory Tasks set at 75 s.
