# Supplementary material for: Ambulatory Tasks and Journeys: A Framework for Free-Living Behaviour
Source: Sensors (Basel). 2026 Mar 10;26(6):1754. doi: 10.3390/s26061754 (PMC13029900; doi:10.3390/s26061754)
Supplement: Supplementary file 1 [file sensors-26-01754-s001.zip › sensors-4122791-supplementary.pdf]

	Individuals (n)	Ambulatory Tasks (hours)	Ambulatory Journeys (minutes)	Sitting (hours)
**All**	3,545	5.78 (2.36)	44.8 (35.9)	9.16 (2.82)
**Sex**				
Male (Reference)	1,669	5.57 (2.48)	46.9 (38.2)	9.54 (2.88)
Female	1,876	5.96 (2.32) *	42.8 (33.5) *	8.83 (2.71) *
**BMI Group**				
Healthy (Reference)	1,140	6.03 (2.31)	48.3 (37.7)	8.86 (2.70)
Overweight	1,352	5.71 (2.34) *	45.9 (36.1) *	9.25 (2.81) *
Obese	886	5.61 (2.36) *	40.3 (33.2) *	9.36 (2.86) *

*Table S1. Baseline Population Characteristics with threshold for Ambulatory Tasks set at 45 seconds. * - Value is significantly different from the reference group*

	Individuals (n)	Ambulatory Tasks (hours)	Ambulatory Journeys (minutes)	Sitting (hours)
**All**	3,545	5.99 (2.43)	32.1 (32.0)	9.16 (2.82)
**Sex**				
Male (Reference)	1,669	5.79 (2.48)	33.4 (33.6)	9.54 (2.88)
Female	1,876	6.61 (2.38) *	30.9 (30.4) *	8.83 (2.71) *
**BMI Group**				
Healthy (Reference)	1,140	6.24 (2.38)	35.3 (34.0)	8.86 (2.70)
Overweight	1,352	5.93 (2.41) *	32.9 (32.3) *	9.25 (2.81) *
Obese	886	5.81 (2.42) *	28.1 (29.0) *	9.36 (2.86) *

*Table S2. Baseline Population Characteristics with threshold for Ambulatory Tasks set at 75 seconds. * - Value is significantly different from the reference group*
